# Biodegradable Polycaprolactone as Ion Solvating Polymer for Solution-Processed Light-Emitting Electrochemical Cells

**DOI:** 10.1038/srep36643

**Published:** 2016-11-04

**Authors:** Nils Jürgensen, Johannes Zimmermann, Anthony John Morfa, Gerardo Hernandez-Sosa

**Affiliations:** 1 Light Technology Institute, Karlsruhe Institute of Technology, Engesserstr. 13, 76131, Karlsruhe, Germany; 2 InnovationLab, Speyererstr. 4, 69115, Heidelberg, Germany

## Abstract

In this work, we demonstrate the use of the biodegradable polymer polycaprolactone (PCL) as the ion solvating polymer in solution-processed light-emitting electrochemical cells (LEC). We show that the inclusion of PCL in the active layer yields higher ionic conductivities and thus contributes to a rapid formation of the dynamic p-i-n junction and reduction of operating voltages. PCL shows no phase separation with the emitter polymer and reduces film roughness. The devices show light-emission at voltages as low as 3.2 V and lifetimes on the order of 30 h operating above 150 cd m^−^^2^ with turn-on times <20 s and current and luminous efficacies of 3.2 Cd A^−1^ and 1.5 lm W^−1^ respectively.

The current rate of technology development has accelerated the rate of acquisition and disposal of electronics that rapidly become obsolete in a matter of several months, causing serious pollution^1^. In 2014 alone 41.8 million tons of electronic waste (e-waste) were produced worldwide^2^ and the number is expected to grow to approximately 65.4 million tonnes by 2017^3^. From this amount of e-waste, only a fraction (<25%) is expected to be recycled^4^.

The research field of green electronics is currently being developed in order to realize the fabrication of biodegradable and biocompatible optoelectronic devices. The development of biodegradable substrates and electrodes, in recent years, demonstrates important and enabling results since these components represent the main bulk of the device^5^,^6^,^7^. These biodegradable devices will enable the use of electronic products that can decompose after their useful lifetime without a negative footprint on the environment^8^,^9^,^10^. The field of organic electronics offers a broad spectrum of tools for the sustainable development of new technologies. Possibilities range from efficient design of functional materials to the use of naturally occurring materials for the fabrication of electronic devices as well as the potential low-cost fabrication of electronic components through high-throughput printing techniques^11^. Problems associated with some organic electronics, such as limited lifetimes^12^, might not apply to applications where biodegradable materials are desirable.

Early work by Irimia-Vladu *et al*.^8^,^10^ demonstrated impressive results from naturally sourced materials such as indigo or beta-carotene for the fabrication of organic-field effect transistors. A promising architecture for the incorporation of biodegradable constituents are organic light-emitting devices that show efficient planar electroluminescence and work on flexible substrates. Applications include smart packaging, which demands economic and ecological components for monitoring or advertising^13^, and custom fit disposable medical devices that can be used for phototherapy^14^. The more common organic light-emitting diode (OLED)^15^ requires functional multi-layer stacks and air-sensitive electron injection materials to reach high performances. To date, electroluminescence of light emitting biomolecules has only yielded small quantum efficiencies, on the order of 10^−7^ 16. However, biodegradable materials could be effectually utilized within OLEDs. For instance, deoxyribonucleic acid was used as an emission layer matrix molecule or as charge blocking layer^17^,^18^. In 2015, Weber *et al*.^19^ used the reemission of different natural fluorescent proteins for successful colour tuning of conventional inorganic light-emitting diodes. Nonetheless, fully biodegradable OLEDs demand biodegradable replacements of various required components to fabricate the complete multilayer architecture.

An advantageous alternative to the OLED is the fabrication of a similar device, the light-emitting electrochemical cell (LEC)^20^. Unlike OLEDs, no reports of LECs with biodegradable constituents have been reported to date. In LECs, the combination of salt ions and emitter molecules form a p-i-n junction *in situ* under an applied bias by electrochemical doping at the electrode interface^21^. Hence, LEC’s active material can consist of only a single layer sandwiched between air stable electrodes. The addition of an ion solvating solid polymer causes a liquid-like environment around the ions, creating the so-called solid polymer electrolyte (SPE), assuring a homogenous ion distribution within the active layer^22^,^23^. Poly(ethylene) oxide is the most commonly used ion solvating polymer^20^. New approaches use oligoethers like the star branched trimethylolpropane ethoxylate derivatives for high performance LECs^24^. Many investigations of SPEs based on biopolymers have been reported, *e.g.* chitosan, gelatine or agar, however processing was done from aqueous solutions whereas the majority of organic emitters are only soluble in non-polar solvents^25^,^26^,^27^.

In this work, we demonstrate the use of polycaprolactone (PCL) as a biodegradable ion solvating polymer for solution-processed LECs. PCL is currently being used as the basis of scaffolds for tissue engineering and drug delivery carrier *in vivo*^28^ and has been proven to be biodegradable by hydrolysis of the backbone ester bonds by enzymes in soil or water^29^,^30^. PCL is soluble in organic solvents and has already shown applicability in SPEs^31^. Herein we show that PCL provides a broad electrochemical stability window (ESW), good ion solvating properties contributing to higher ionic conductivities in the active material and thus a rapid formation of the dynamic p-i-n junction and reduction of operating voltages. Solution-processed LEC devices show light-emission at voltages as low as 3.2 V and lifetimes on the order of 30 h operating above 150 cd m^−^^2^, demonstrating that PCL can be used as an ion solvating polymer.

## Results and Discussion

An important aspect for the LEC’s device performance is the electrochemical stability of the SPE. Irreversible side reactions of the SPE under device operation can hinder the doping of the organic semiconductor and adversely affect device characteristics^32^. Thus, the long term operational stability of LECs is greatly dependent on the ESW of PCL and whether or not it encloses the redox potentials of the emitting semiconductor, in the present case a commercial PPV derivative known as Super Yellow (SY)^33^.

**Figure 1:**
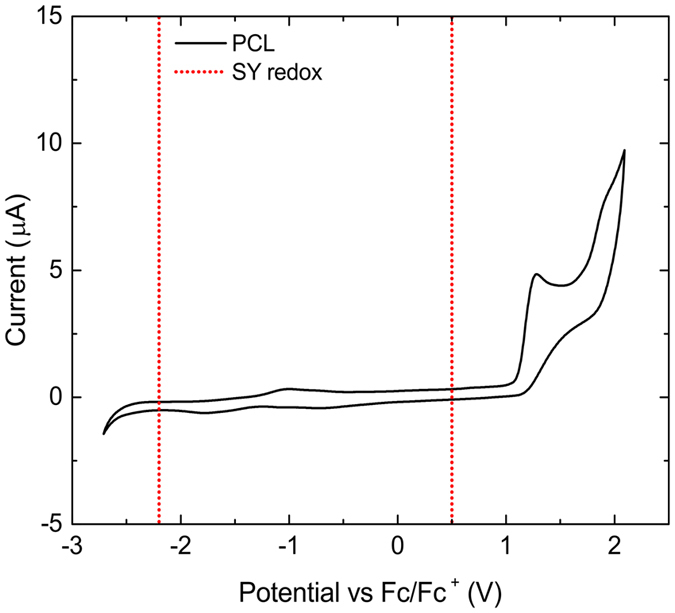
Cyclic voltammogram of PCL in acetonitrile with TBAPF_6_ as the supporting electrolyte vs. Fc/Fc^+^. PCL irreversibly oxidizes at 1.1 V and shows no reduction within the ESW of TBAPF_6_. The red dotted lines mark the SY redox potentials 0.5 V and −2.2 V^34^.

Figure 1 shows the cyclic voltammogram of PCL in acetonitrile as well as the oxidation (p doping) and reduction (n doping) potentials of SY as reported in literature *vs.* Ferrocene (Fc/Fc^+^)^34^. Tetrabutylammonium hexafluorophosphate (TBAPF_6_) was used as the supporting electrolyte. PCL shows an irreversible oxidation peak with an onset of 1.1 V and no reduction reaction in between the ESW of TBAPF_6_, so the reduction potential of PCL should lie at lower voltages. Hence, the ESW of PCL is wide enough to allow doping of SY without degrading.

**Figure 2:**
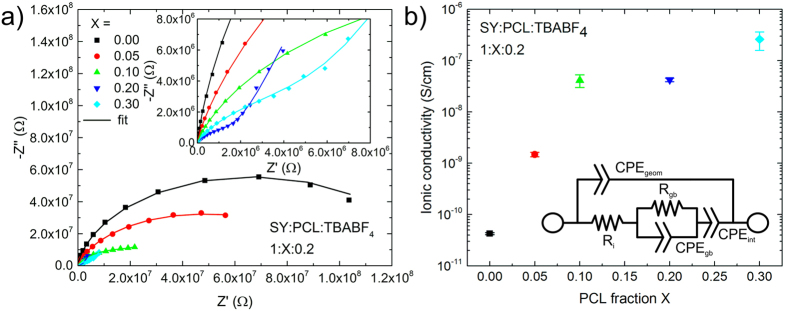
Impedance spectroscopy measurement of pristine SY:PCL:TBABF_4_ LECs show increased ionic conductivity with PCL ratio. (**a**) Nyquist plots of a representative device measured with a 50 mV AC signal and 0 V DC bias. The inset shows an enlargement of the measurements with higher ionic conductivity. (**b**) Calculated ionic conductivity with s.d. of two pristine pixels on two different substrates (four total devices). The inset shows the equivalent circuit diagram^36^ used to fit the Nyquist plots in (**a**), where *R_i_* models the ionic resistance.

In order to investigate the ionic conductivity of PCL in sandwich device LECs, we studied a series of pristine LECs of two pixels on two different substrates (four total devices) with an active layer composed of PCL and tetrabutylammonium tetrafluoroborate (TBABF_4_) as the SPE blended with the light-emitting SY. Though, the authors do not know of any studies of the biodegradability of SY and TBABF_4_, the use of these materials will enable benchmarking of the performance of PCL to known blends. We measured the samples by impedance spectroscopy at 50 mV AC and 0 V DC bias, with typical results shown in Fig. 2. Munar *et al*. have shown that this technique is an appropriate method for determining key processes including the ionic conductivity^35^. The equivalent circuit consists of a constant phase element (CPE) representing the geometric capacitance of the electrodes *CPE*_*geom*_ in parallel to the ionic resistance *R*_*i*_ and the electrode-interlayer capacitance *CPE*_*int*_ due to the electric double layers. In our work, we extended this model by a parallel *R*_*gb*_-*CPE*_*gb*_-circuit considering grain boundary effects of phases with different ionic mobility suggested by Huggins *et al*.^36^. The full circuit can be seen in the inset of Fig. 2(b). Figure 2(a) shows the Nyquist plots for a typical device at each of the varying PCL fractions, *X*, with constant fractions 1 and 0.2 for SY and TBABF_4_ respectively of each fraction. By fitting the equivalent circuit, we could extract the ionic resistance *R*_*i*_. All parameters of the shown fits can be seen in Supplementary Table S1. With an active area *A* = 24 mm^2^ and the measured thicknesses *d*, see Table 1, we calculated the ionic conductivity using *σ*_*i*_ = *d* (*AR*_*i*_)^−1^, which is shown in Fig. 2(b). It is clear that the inclusion of PCL increased the ionic conductivity in the active layer by up to four orders of magnitude compared to the sample with no PCL, reaching a value of 2.6 10^−7^ S cm^−1^ for 0.3 parts of PCL. This result stands in very good agreement to comparable studies, where ionic conductivities of up to 10^−4^ S cm^−1^ for pure PCL-based SPEs were measured. Therefore, this improvement can be accredited with a more facile movement of the TBABF_4_ ions dissolved within the PCL and SY blend.

The influence of the enhancement of the ionic conductivity by PCL on the device operational parameters were determined with luminance-current-voltage (LIV) measurements. Table 1 presents the LIV characteristic’s average of again two pristine pixels on two different substrates (four total devices), each using a voltage sweep rate of 100 mV s^−1^. The correspondent LIV curves can be seen in Supplementary Figure S1. It was observed that increasing the PCL content, while keeping a constant SY:TBABF_4_ ratio of 1:0.2, leads to a reduction of the turn-on voltage from 5.5 V to 3.2 V suggesting a more effective dynamic formation of the p- and n-doped region within the devices due to the improved ionic mobility. The turn-on voltage was defined as the voltage needed to achieve a luminance greater than 1 cd m^−2^. The device current efficacy and luminous efficacy for 100 and 1000 cd m^−2^ improved and, in addition, the turn-on behaviour was more consistent with PCL. It is important to point out that the SY:TBABF_4_ ratio of 1:0.2 was chosen because it showed the lowest turn-on voltage compared to 0.1 or 0.3 parts of TBABF_4_, which can be seen in Supplementary Figure S1. A Supplementary Video of the operating devices and the derivative of the luminance voltage characteristic (see Supplementary Figure S2) can be seen.

**Figure 3:**
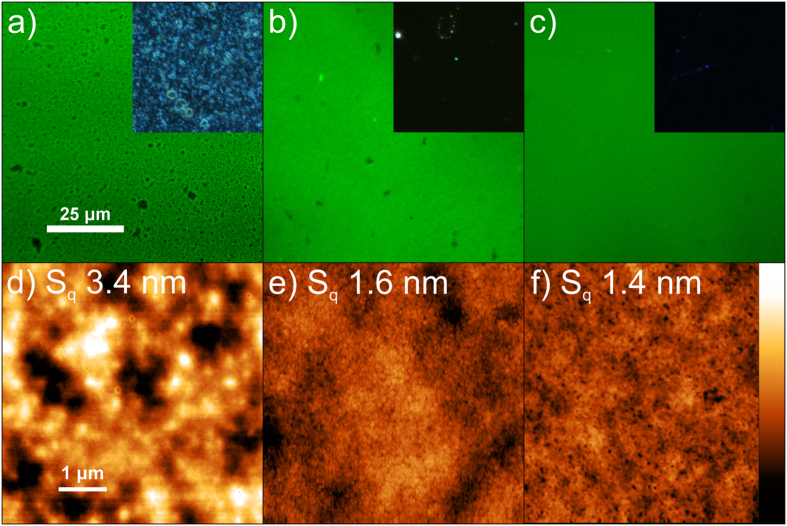
Photoluminescence microscopy, dark field optical microscopy (inset) (**a–c**) and AFM pictures (**d–f**) of SY:PCL:TBABF_4_ films on ITO and glass respectively with the ratios 1:0.00:0.2 (**a,d**), 1:0.05:0.2 (**b**,**e**) and 1:0.10:0.2 (**c,f**). The maximum height of the AFM profiles is *z*_0_ = 20 nm and the area root mean squared roughness *S*_*q*_ of the AFM pictures is shown in the pictures.

For a more concrete understanding of the device operation, we characterized the light-emitting layers. The film morphology and, in particular, the intermixing of the SPE and the emitter material have been reported to be crucial to LEC device performance^37^,^38^,^39^. Different parameters such as crystallization, molecular weight, hydrophobicity or hydrophilicity can affect the miscibility of the SPE and the emitter. Phase separation of the two components will lead to an inhomogeneous distribution of ions within the film, affecting the efficiency of p-i-n junction formation, and in addition, creating regions in which the lack of emitter material will result in no light emission. A comparison of the film morphology between the different active layers is presented in Fig. 3 by means of photoluminescence (PL), dark field and atomic force microscopy (AFM). Figure 3(a) presents the PL image of a SY:TBABF_4_ film with 1:0.2 weight ratio. The image shows the fluorescent regions where the SY emission is observable as well as darker regions, which suggest the presence of TBABF_4_ agglomerates. A dark field microscopy image of the same region on the sample is presented in the corresponding inset. The image indicates the existence of a broad number of light-scattering features. This inhomogeneous distribution of the salt supports the supposition that the low ionic mobility and consequently the lower device performance compared to samples with PCL inside arises from poor salt solubility in SY. The microscopy images of samples with 0.05 and 0.1 parts of PCL are presented in Fig. 3(b,c) respectively. The PL microscopy images present a homogeneous fluorescence distribution with only a few small agglomerates while the dark field images show the almost complete removal of light scattering agglomerates. In this case, the ion solvating capabilities of PCL lead to a complete dissolution of the TBABF_4_ agglomerates resulting in a, presumably, uniform ion distribution throughout the full active layer. Furthermore, SY and PCL completely intermix and do not form phase separation even for higher parts of up to 0.3 parts of PCL as shown in Supplementary Figures S3 and S4. Consequently, the favourable miscibility of PCL with SY and the improved salt distribution is accompanied by a higher mobility of the ions, which resulted in the observed reduction of the operational voltage.

AFM images of SY with only salt and with salt and 0.05 or 0.1 parts PCL are shown in Fig. 3(d–f) respectively. The area root mean squared roughness *S*_*q*_ of the sample with salt and without PCL *S*_*q*_ = 3.4 nm has drastically increased in comparison to pristine SY films with *S*_*q*_ = 0.4 nm as shown in Supplementary Figure S5. This higher roughness can be traced back to the salt agglomerates. By adding PCL, the surface smooths, reducing the roughness to *S*_*q*_ = 1.6 nm and *S*_*q*_ = 1.4 nm for 0.05 and 0.1 parts of PCL, respectively, due primarily to the solvation of the ions (*i.e.* loss of salt aggregates).

**Figure 4:**
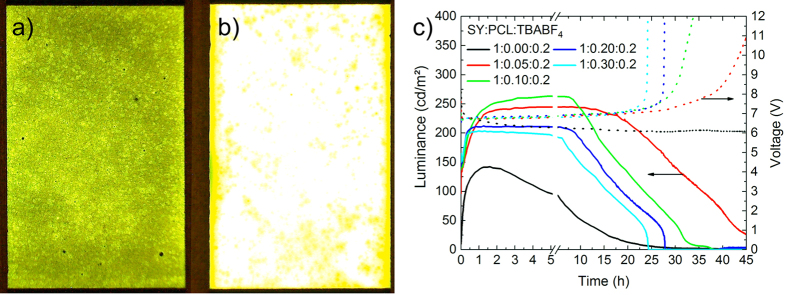
Emitting 4 mm by 6 mm pixels of SY:PCL:TBABF_4_ LECs without (**a**) and with 0.05 parts PCL (**b**) at 5 s of the lifetime measurement shown in (**c**). Current-voltage-luminescence-time (**c**) lifetime measurement of LECs with different PCL concentrations. The curves were recorded with pulsed current driving scheme (*J* = 8.33 mA cm^−2^) at 1000 Hz with 50% duty cycle. The solid and dashed lines represent the evolution of the luminance and the operational voltage, respectively.

Finally, the effect of PCL on the LEC device performance was determined. Figure 4 shows the lifetime characteristics of the LECs with the same examined ratios as above. The devices were pulsed current driven at a current density of *J* = 8.33 mA cm^−2^ and a frequency of 1000 Hz with a 50% duty cycle after a linear 4 s current ramp to establish the operating current density. A visual comparison of a pixel without (Fig. 4a) and with 0.05 parts PCL (Fig. 4b) at 5 s after start of the pulsed lifetime measurement clearly shows the faster turn-on behaviour. The pixel with PCL appears brighter as it turns on faster. This is also seen in Fig. 4(c), which shows the luminance and voltage of the LECs plotted over time. The lifetime (*t*_*50*_) was defined as the time taken for the luminance to drop to half of the maximum achieved luminance, which was accompanied by the respective increase in operational voltage as the device deteriorates. We determined that the sample containing no PCL reached a lifetime of *t*_*50*_ ≈ 7 h and maximum luminance *L*_*max*_ = 142 cd m^−2^.

The inclusion of the 0.05 parts of PCL was observed to extend the device lifetime four fold to *t*_*50*_ ≈ 32 h and exhibited a *L*_*max*_ = 245 cd m^−2^. A further increase in the PCL content to 0.1 achieved a higher *L*_*max*_ = 262 cd m^−2^, however, reduced the lifetime to *t*_*50*_  ≈ 20 h. Additional inclusion of PCL was observed to further decrease the operational lifetime down to *t*_*50*_ ≈ 16 h and the maximum luminance to *L*_*max*_ = 202 cd m^−2^ for the sample with 0.3 parts of PCL. The turn-on time (*t*_*on*_) was defined as the time to reach 100 cd m^−2^. The device without PCL showed a turn-on time of *t*_*on*_ = 16 min. 0.05 parts of PCL already reduced the turn-on time to *t*_*on*_ = 23 s. Further increase in PCL ratio lead to turn-on times *t*_*on*_ < 20 s. PCL also showed a positive influence on the current efficacy and luminous efficacy. The device with 0.1 parts PCL reached a maximum of 3.2 Cd A^−1^ and 1.5 lm W^−1^ in current and luminous efficacy respectively whereas the device without PCL reached 1.7 Cd A^−1^ and 0.8 lm W^−1^. The equivalent chronological sequence can be seen in Supplementary Figure S6. After lifetime testing, visual inspection of the pixels showed that the characteristic yellow colour of SY had vanished from the active area of all devices containing PCL. This discoloration and the voltage increase suggest a destruction of the conjugated polymer by electrochemical reactions with TBABF_4_ which seems to limit the performance as indicated in the cyclic voltammogram of TBABF_4_ in Figure S7. These undesired electrochemical reactions with TBABF_4_ may be the reason why the lifetime decreases with increasing the PCL content above 0.05 parts. As the overall amount of SY in the active layer decreases with increasing PCL content, the reaction with SY likely occurs faster when there is less SY inside. However, PCL leads to an efficient increase in lifetime, maximum luminance and turn-on time of LEC devices.

Taking everything into account, LECs with PCL could not reach performances of state of the art LECs with several hundred hours in lifetime or above 10 cd A^−1^ or lm W^−1^ in current and luminous efficacy respectively^24^ but most importantly showed very fast turn-on times and a homogenous light distribution. In addition, the broad ESW of PCL enables options of combining PCL with even higher bandgap emitter materials. Considering device preparation and operation in ambient conditions, the reached lifetimes of 30 h fulfil the demands of biodegradable short use devices.

## Conclusions

In summary, we demonstrated the use of the biodegradable polymer PCL as ion solvating polymer in the active layer of solution-processed LECs. PCL showed good miscibility with the polymeric emitter and excellent ion solvating capability allowing for faster turn-on times and increased operational lifetimes. Nevertheless, further use of more electrochemically stable salts should overcome this limitation. The present demonstration of the use of biodegradable polymers as part of the emitter layer for luminescent devices is an important step for pushing the boundaries of biodegradable components. Future research efforts will be aimed at the replacement of the conjugated polymer and salt by biodegradable counterparts in order to achieve a completely bio emission layer. Fully biodegradable devices can contribute to a sustainable production of consumption electronics and furthermore access new pathways for biocompatible electronics research.

## Methods

### Materials

All the materials to produce the devices were purchased and used as received. Super Yellow (SY, Livilux PDY-132) was purchased from Merck. Polycaprolactone (PCL, *M*_*w*_ = 14 000 g mol^−1^), tetrabutylammonium tetrafluoroborate (TBABF_4_, ≥99%) and anisole (≥99%) were obtained from Sigma Aldrich. Pre-structured Indium Tin Oxide (ITO) coated electronic grade glass (180 nm, 10 Ω □^−1^) was acquired from Kintec.

### LEC Fabrication and Characterization

SY, PCL and TBABF_4_ were separately dissolved in anisole solutions with a concentration of 10 g l^−1^ that were intermixed at the relevant weight ratios and shaken for 24 h. ITO substrates were cleaned in an ultrasonic bath for 10 min in acetone and isopropanol respectively followed by 5 min oxygen plasma treatment. The SY:PCL:TBABF_4_ active layers were spin-coated in two steps at 1700 rpm for 45 s and 3500 rpm for 10 s and annealed in a vacuum oven at 40 °C and 10 mbar for 2 h afterwards. A 100 nm silver electrode was thermally evaporated through a shadow mask at 10^−6^ mbar yielding a 24 mm^2^ active area.

For LEC characterization, calibrated Botest LIV functionality and OLT lifetime test systems were used. Luminance-current-voltage (LIV) characterizations were measured with a 100 mV s^−1^ sweeping rate. Lifetime (LT) data was collected in a pulsed current mode at 8.33 mA cm^−2^ and 1000 Hz with a 50% duty cycle after a linear 4 s current ramp to establish the operating current density. The pictures of the emitting pixels were taken with a dnt DigiMicro Scale.

Impedance spectroscopy measurements were carried out on an Autolab PGSTAT302N potentiostat. A 50 mV AC voltage signal was used to measure the device impedance with the frequency varying from 10^4^ to 0.05 Hz at 0 V bias.

### Film Characterization

Optical microscopy pictures were taken with a Nikon Eclipse 80i microscope with LU Plan Fluor 50x objectives and an Intensilight C HGFI Pre-centered Fiber Illuminator with an 380–600 nm mercury lamp and an EPI-FL B 2E/C filter for fluorescence microscopy. Atomic force microscopy was done with a Semilab DME Compact Granite DS 95 SPM Head. Film thicknesses were determined using a Veeco Dektak 150 profilometer.

### Cyclic Voltammetry

Platinum working and counter electrodes were used in combination with a silver wire reference electrode *vs.* ferrocene Fc/Fc^+^ in acetonitrile (>99.9%, Sigma Aldrich) with 0.1 mol l^−1^ tetrabutylammonium hexafluorophosphate (TBAPF_6_, ≥99.0%) as the supporting electrolyte under nitrogen atmosphere. The measurements were performed using an Autolab PGSTAT302N potentiostat.

## Additional Information

**How to cite this article**: Jürgensen, N. *et al*. Biodegradable Polycaprolactone as Ion Solvating Polymer for Solution-Processed Light-Emitting Electrochemical Cells. *Sci. Rep.* **6**, 36643; doi: 10.1038/srep36643 (2016).

**Publisher’s note:** Springer Nature remains neutral with regard to jurisdictional claims in published maps and institutional affiliations.

## Supplementary Material

Supplementary Information

Supplementary Information

## Figures and Tables

**Table 1:** LIV Characteristics of SY:PCL:TBABF_4_ (1:X:0.2) LECs measured at 100 mV s^−1^ with s.d. of two pristine pixels on two different substrates (four total devices).

PCL Content	thickness *d*(nm)	turn-on voltage.a (V)	max luminance (cd m^−^^2^)	current efficacy (cd A^−1^)		luminous efficacy (lm W^−1^)	
				100 cd m^−^^2^	1000 cd m^−^^2^	100 cd m^−^^2^	1000 cd m^−^^2^
0.00	123 ± 8	5.5 ± 0.2	7544 ± 460	0.21 ± 0.02	0.31 ± 0.02	0.09 ± 0.01	0.13 ± 0.01
0.05	143 ± 7	4.1 ± 0.1	4134 ± 379	0.37 ± 0.04	0.23 ± 0.02	0.21 ± 0.02	0.11 ± 0.01
0.10	142 ± 9	3.8 ± 0.1	3820 ± 178	0.46 ± 0.01	0.21 ± 0.01	0.28 ± 0.01	0.11 ± 0.01
0.20	136 ± 5	3.2 ± 0.1	3793 ± 140	1.31 ± 0.21	0.45 ± 0.04	1.01 ± 0.18	0.28 ± 0.01
0.30	125 ± 6	3.3 ± 0.1	4146 ± 95	0.93 ± 0.03	0.49 ± 0.02	0.68 ± 0.02	0.29 ± 0.01

^a^voltage at 1 cd m^−2^.
