# Supplementary material for: Biodegradable Polycaprolactone as Ion Solvating Polymer for Solution-Processed Light-Emitting Electrochemical Cells
Source: Sci Rep. 2016 Nov 4;6:36643. doi: 10.1038/srep36643 (PMC5095640; doi:10.1038/srep36643)
Supplement: Supplementary Information [file srep36643-s1.pdf]

Biodegradable Polycaprolactone as Ion Solvating Polymer for Solution-Processed Light-Emitting Electrochemical Cells

Nils Jürgensen[1,2], Johannes Zimmermann[1,2], Anthony John Morfa[1,2] & Gerardo Hernandez-Sosa[1,2]*

[1]Light Technology Institute, Karlsruhe Institute of Technology, Engesserstr. 13, 76131, Karlsruhe, Germany

[2]InnovationLab, Speyererstr. 4, 69115, Heidelberg, Germany

*corresponding author: gerardo.sosa@kit.edu

1. Ionic Conductivity

2. Luminance-current-voltage (LIV)

3. Microscopy

4. Lifetime

5. Cyclic Voltammetry

6. Video

1. Ionic Conductivity

PCL content	R_i (Ω)	R_{gb} (Ω)	CPE_{geom}		CPE_{int}		CPE_{gb}	
			Q	n	Q	n	Q	n
0.00	$1.2 \cdot 10^{6}$	$136 \cdot 10^{6}$	$5.4 \cdot 10^{-9}$	1	$1.1 \cdot 10^{-7}$	0.6	$4.6 \cdot 10^{-9}$	0.7
0.05	$46.6 \cdot 10^{3}$	$87 \cdot 10^{6}$	$5.1 \cdot 10^{-9}$	1	$6.3 \cdot 10^{-7}$	1.0	$13.1 \cdot 10^{-9}$	0.7
0.10	$1.6 \cdot 10^{3}$	$22 \cdot 10^{6}$	$5.0 \cdot 10^{-9}$	1	$2.6 \cdot 10^{-7}$	0.6	$32.6 \cdot 10^{-9}$	0.7
0.20	$1.2 \cdot 10^{3}$	$2 \cdot 10^{6}$	$6.2 \cdot 10^{-9}$	1	$3.9 \cdot 10^{-7}$	0.8	$113.0 \cdot 10^{-9}$	0.6
0.30	143	$6 \cdot 10^{6}$	$6.2 \cdot 10^{-9}$	1	$3.1 \cdot 10^{-7}$	0.8	$96.1 \cdot 10^{-9}$	0.6

Table S1. Parameters of the fits within the Nyquist plots of Figure 2 a). The impedance of a *CPE* is given by $Z_{CPE} = 1/(Q(\omega i)^n)$ with the frequency ω, the imaginary number *i* and frequency independent numerical parameters *Q* and *n* ($0 \geq 1$).

2. LIV

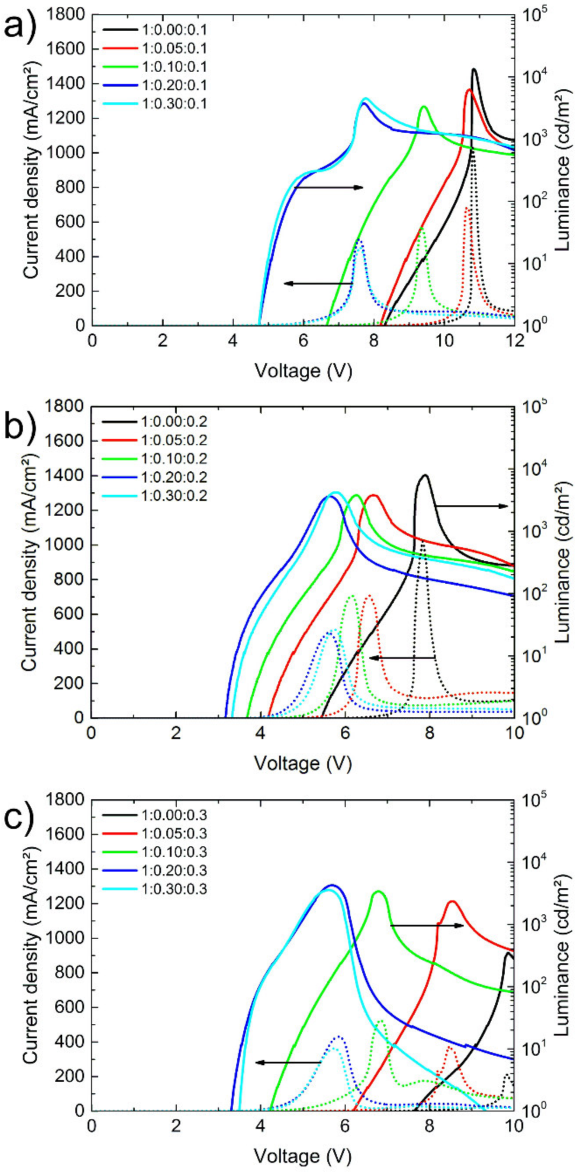

Figure S1. LIV characteristic of SY:PCL:TBABF$_4$ LECs with 0.1 a), 0.2 b) and 0.3 c) parts of TBABF$_4$ for different PCL contents swept at 100 mV s^{-1}. The devices turn on at lower operating voltages and the current density reduces with rising PCL content.

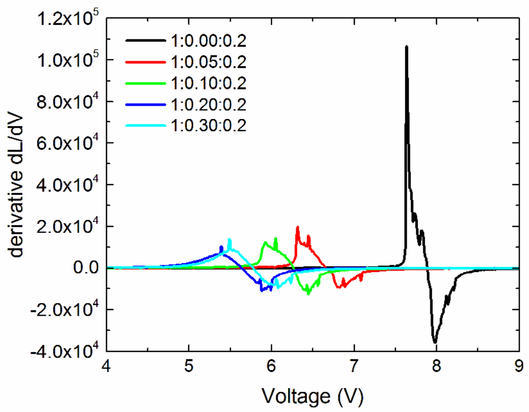

Figure S2. Derivative of the LV characteristic shown in Figure S1. With increasing PCL content the turn-on behavior becomes more consistent before degradation. The device without PCL shows a steep gain and an immediate degradation in comparison to the devices with PCL.

3. Microscopy

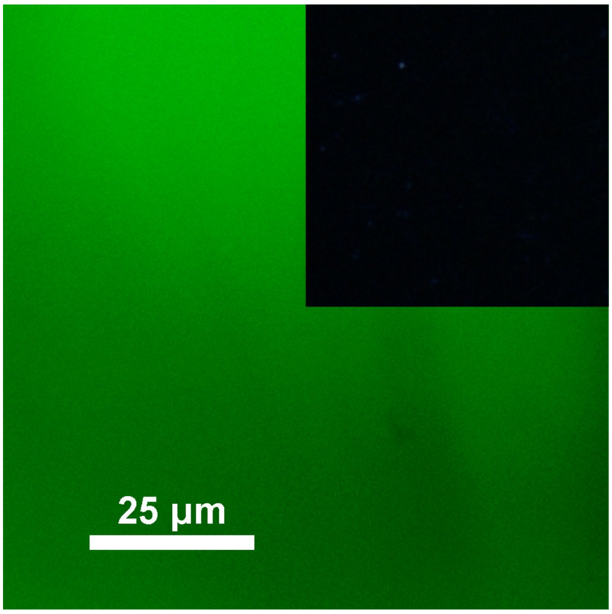

Figure S3. PL and dark field (inset) of SY:PCL:$TBABF_4$ 1:0.3:0.2. The PL picture shows no phase separation of SY and PCL and only a few isolated scattering particles can be seen in the dark field picture.

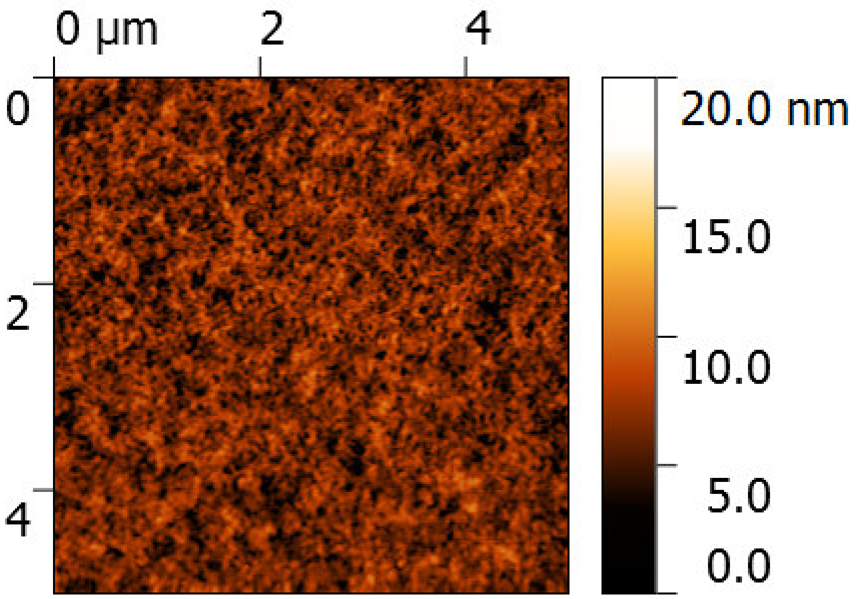

Figure S4. AFM image of SY:PCL:$TBABF_4$ 1:0.3:0.2 on glass, S_q = 1.4 nm.

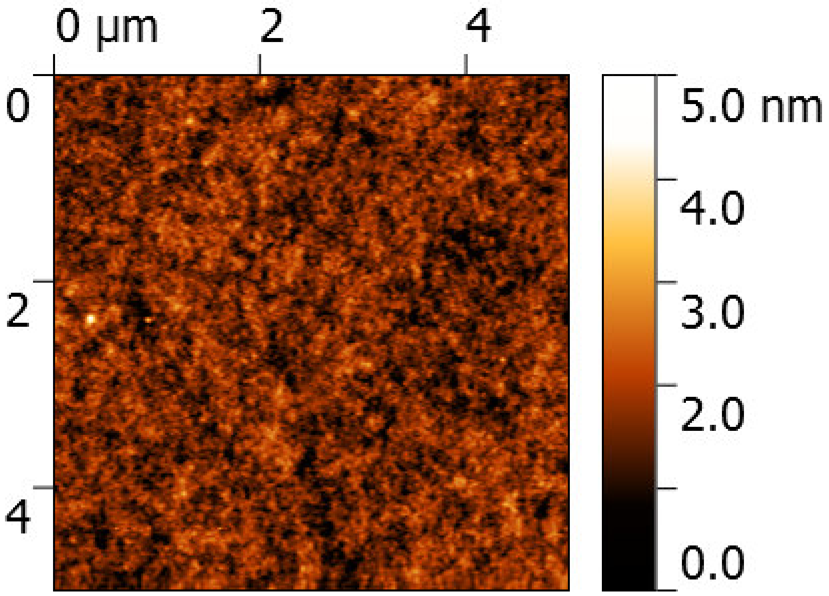

Figure S5. AFM picture of SY film on glass, S_q = 0.4 nm.

4. Lifetime

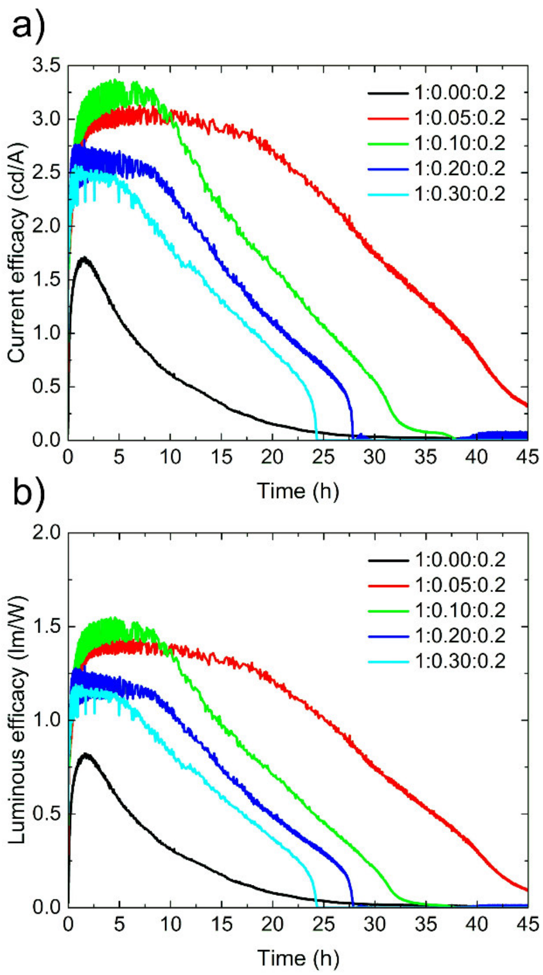

Figure S6. Trends of a) current efficacy and b) luminous efficacy of the lifetime measurements over time of the SY:PCL:$TBABF_4$ LEC devices. The devices with 0.10 parts PCL showed the highest current and luminous efficacy with two times higher values than the devices without PCL.

5. Cyclic Voltammetry

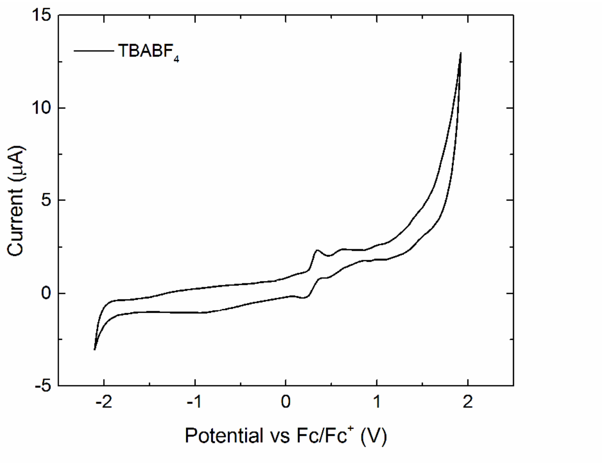

Figure S7. CV of $TBABF_4$ in acetonitrile *vs Fc/Fc⁺*. $TBABF_4$ shows irreversible oxidation and reduction reactions above 1 V and below -2 V respectively. A reversible redox reaction occurs at 0.3 V which is comparable small to the aforementioned oxidation and reduction reactions.

6. Video

The video "Emission comparison of LECs containing PCL" was taken with a Canon EOS 6d with a Canon EF 100mm f/2.8 Macro objective. It shows the 4 mm x 6 mm emission areas of light-emitting electrochemical cells with rising PCL content.
